# The prevalence of thyroid nodules and its factors among Chinese adult women: A cross-sectional study

**DOI:** 10.3389/fendo.2022.967380

**Published:** 2022-08-17

**Authors:** Xiaoqian Dong, Ying Li, Jianfei Xie, Lijun Li, Ziyu Wan, Yue Kang, Yating Luo, Jiangang Wang, Yinglong Duan, Siqing Ding, Andy SK Cheng

**Affiliations:** ^1^ Health Management Center, The Third Xiangya Hospital of Central South University, Changsha, China; ^2^ Xiangya Nursing School, Central South University, Changsha, China; ^3^ Nursing Department, The Third Xiangya Hospital of Central South University, Changsha, China; ^4^ Emergency Department, The Third Xiangya Hospital of Central South University, Changsha, China; ^5^ The Hong Kong Polytechnic University, Department of Rehabilitation Sciences, Hong Kong, Hong Kong SAR, China

**Keywords:** thyroid nodule, lifestyle, metabolic syndrome, adult women, China

## Abstract

**Objective:**

To determine the prevalence of thyroid nodules in Chinese adult women. To analyze the relationships between lifestyle, metabolic syndrome and thyroid nodules.

**Methods:**

We conducted a retrospective cross-sectional study in the tertiary hospital from 2017 to 2019. Included participants underwent thyroid color Doppler ultrasonography, lipids examination, and dietary evaluation.

**Results:**

Totally 2,784 participants were included, and 933 participants were found to have thyroid nodule(s) by B-ultrasound. The prevalence of thyroid nodules was 33.3%. Women in 50-59 years (OR: 1.746, 95% CI [1.356-2.249]), older than 60 (2.147 [1.540-2.993]) and occupations with mainly manual work (1.780 [1.367-2.317]) were risk factors for thyroid nodules, while moderate dietary diversity (0.624 [0.476-0.817]) and normal triglycerides level (0.739 [0.604-0.905]) were protective factors.

**Conclusion:**

Women over 50 and those whose jobs are mainly manual should enhance screening, follow-up and health management of thyroid nodules. Higher dietary diversity is protective measures against thyroid nodules for adult women and should consider dietary balance and the food varieties, not just increased quantities.

## Introduction

Thyroid nodule (TN), the common clinical thyroid disease with a prevalence of 19%-68% (1), has become increasingly prevalent worldwide. They are the sporadic lesion caused by abnormal local growth of thyroid cells and 5%-10% of patients with TN(s) will become malignant thyroid cancer (TC) (2), which is the most common endocrine malignancy worldwide and ranks ninth (3) on the tumor incidence scale. It is also the second most common cancer in young women. The annual percentage change of the incidence of thyroid cancer in Chinese women has increased from 4.9% in 2000-2003 to 20.1% in 2003-2011 (4). However, most TN(s) show few typical symptoms and signs, and are not easily touched by hands, so they are neglected and best treatment opportunities are missed likely.

Recent studies reported that BMI was a risk factor for TN(s) (5–7), especially in elderly individuals. However, smoking, drinking, exercise and Mediterranean diet may have protective effects on TN(s) (6, 8, 9). But these lifestyle factors were usually studied separately, and some of these factors seem to be contradictory (5, 6). A complete healthy lifestyle includes a varied diet, good eating habits, regular physical exercise, moderate alcohol consumption and non-smoking (8, 10–12). Dietary factors are influenced by different eating patterns and habits, nutrition and other environmental factors (13). Currently, iodine deficiency or adequacy have been considered risk factors for thyroid disease and are closely associated with the development of TC, but the details of different food types have been controversial in previous studies. In general, diets are multi-dimensional exposures, with thousands of ingredients in foods, beverages consumed and dietary intake varying daily, and can not be accurately measured in free-living populations (14). Dietary Diversity Score (DDS) is *a priori* defined dietary quality evaluation index, which only counts the types of food intake, regardless of frequency and intake (15). Dietary Diversity is indicated to high diet quality for different populations (16, 17) and an important part to healthy diet (15). It is significantly and independently associated with goiter, and children with poor DDS are almost twice as likely as their peers to develop goiters (18). But no studies have been done on adult women.

The relationships between physical activities and health or disease are complex and unpredictable. The 2018 Physical Activity Guidelines for Americans (PAGA) and the World Cancer Research Fund/American Institute for Cancer Research (WCRF/AICR) assessed and summarized epidemiological evidence on the association between physical activity and cancer risk as the part of recommendations on physical activity to reduced cancer risk (8). Specifically, there was strong evidence that physical activities reduced cancer risk, and high and low levels of sedentary time were consistently associated with risk range (12). But this summary of evidence did not include the TC study. A case-control study in southern Italy showed that daily walking time was closely associated with a reduced risk of TC (19). However, an exercise program of twelve weeks had no significant effect on hormones related to thyroid function and blood lipids (20). Although the health benefits of physical activity are well established, the relationship between physical activity and thyroid disease remains to be explored.

It has been confirmed that it is closely related to age and gender and other factors (21). The incidence of TN(s) in women is significantly higher than that in men (22–24), but the specific mechanism is not completely clear. It may be associated with overweight or obesity, hypertension, hyperglycemia, metabolic syndrome, sleep quality, etc. (3). It is worth noting that most of these risk factors are components of Metabolic Syndrome (MetS), which includes abdominal obesity; atherogenic dyslipldemia; hypertension; hyperglycemia and so on. A meta-analysis showed that a strong correlation between TN(s) and MetS in the non-iodine-deficient population (25). A study found that the thyroid was the target organs for MetS (3), and a portion of MetS patients had thyroid nodules (26). Previous studies had shown that obesity was an independent risk factor for TN(s) (27), especially central obesity, but no association between overweight and hypothyroidism had been observed (28). On the other hand, a meta-analysis showed that central obesity showed gender differences and was a risk factor for thyroid nodules in men but not in women (29). Hypertension, fasting glucose and diabetes were associated with a higher risk of TN(s) (30). A multi-center survey also showed significant differences in body mass index and triglycerides between the TN(s) group and the non-TN(s) group (31), which was inconsistent with another survey (26). Thus, the relationship between TN(s) and main components of MetS remains questionable.

At present, high resolution ultrasound is widely used in the screening and evaluation of TN(s), which has advantages in the qualitative, localization and quantitative diagnosis of thyroid tumors that other imaging examinations do not have. The prevalence of TN(s) by palpation is 4-7%, while the prevalence by ultrasound is 13-67% (32). However, the implementation of thyroid ultrasound screening strategies among people in China is still not widespread, so there is a lack of such data and their relevance to individual lifestyles.

This cross-sectional study was to determine the prevalence of TN(s) among China adult women and the influence of lifestyle and components of MetS on TN(s) as well as their predictive factors.

## Materials and methods

### Study design

**Figure 1:**
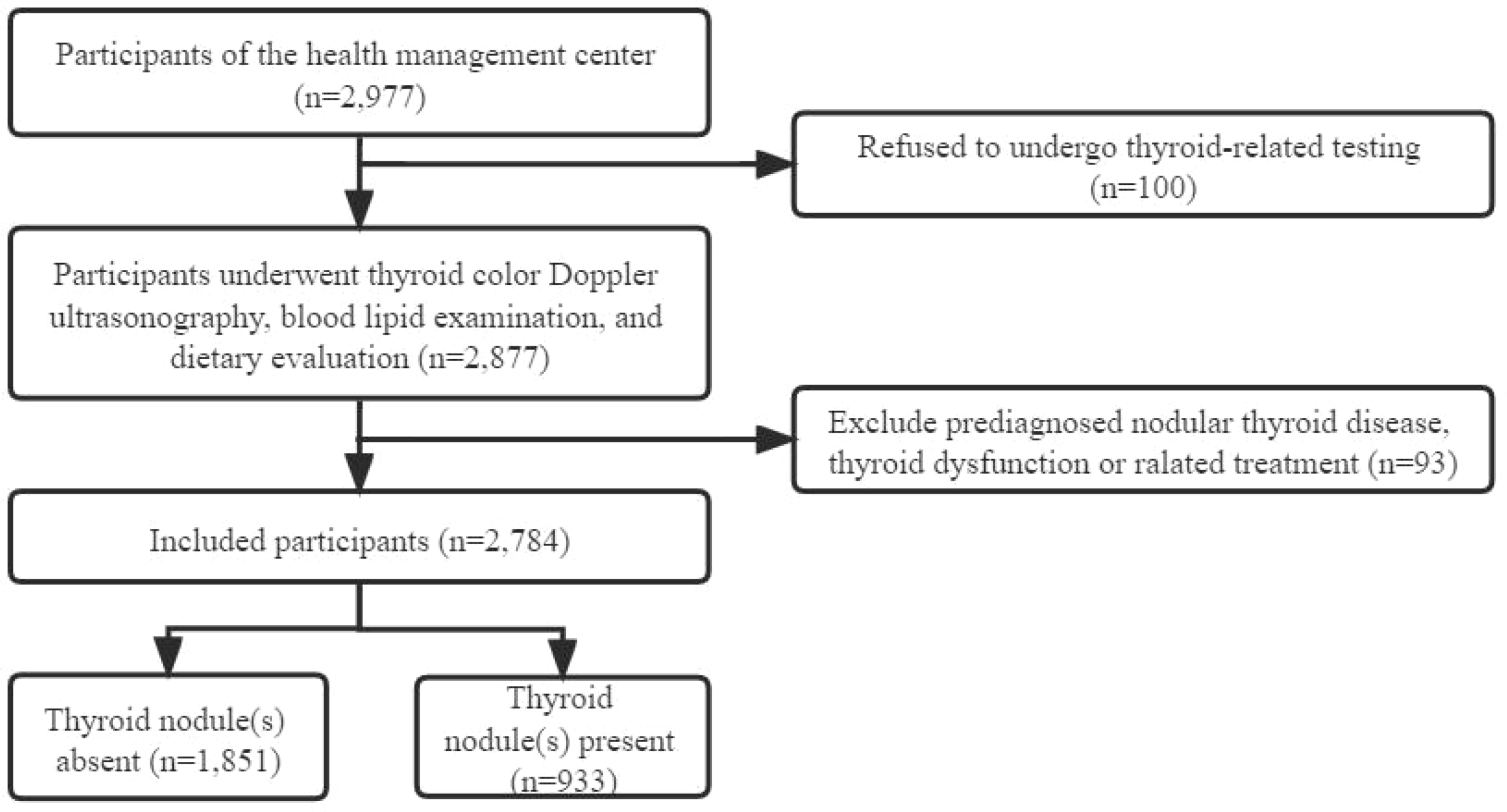
Flowchart of this study.

We conducted a cross-sectional study and recruited participants from the health management center of general tertiary hospital located in southern China between 1 January 2017 and 31 December 2019. The inclusion criteria were: (1) women over the age of 18; (2) underwent thyroid color Doppler ultrasonography, blood lipid examination, and dietary evaluation; (3) voluntary participation in this study. The exclusion criteria were: (1) known thyroid disease, such as pre diagnosed nodular thyroid disease, thyroid dysfunction or under treatment with thyroid hormone or anti-thyroid drugs followed up by endocrinologists; (2) a history of previous neck radiation therapy (see **Figure 1**).

### Demographic characteristics

We collected age and occupations from each participant. We divided the age into four categories: < 40 years old, 40-49 years old, 50-59 years old and ≥ 60 years old. Based on national physical labor intensity classification, we defined occupations as: brain worker, manual worker, brain and manual worker, and retiree.

### Lifestyle assessment

Lifestyle was divided into dietary habits, exercise and sedentary behavior. Eight components of dietary habits were rated: (1) BMI; (2) the dietary diversity scale; (3) midnight snacks; (4) social engagement; (5) coffee; (6) sugar-sweetened beverages; (7) smoking; and (8) alcohol consumption.

BMI was calculated by dividing body mass (kg) by height (m) square (BMI = kg/m2). It (33) was categorized as lean, normal weight, overweight, and obese for BMI < 18.5 kg/m^2^, 18.5-23.9 kg/m^2^, 24.0-27.9 kg/m^2^, and ≥ 28.0 kg/m^2^, respectively.

Dietary Diversity Scale (DDS) (34) is based on a balanced diet pagoda, which divides foods into nine categories: grains (including cereals, roots, and tubers), vegetables, fruits, livestock meat (including pork, poultry, beef, and organs), fish and shrimp (including seafood, freshwater fish, and aquatic products), eggs, milk, and dairy products, beans (including beans, nuts, and seeds), and oils and fats (including animal and vegetable oil). Food other than the above mentioned nine food categories, such as carbonated drinks, tobacco, coffee, etc., were not included in this score. DDS was calculated by adding up the total number of types of food each participant consumed in the past week, regardless of the frequency or quantity of food consumed. If a participant consumed any of the mentioned foods, he would receive one point in that food category; otherwise, he would receive zero points. Consumption of different foods from the same category will not be counted. The total score can be up to 9 points, which is categorized to three degrees as follows: 1-5 points as insufficient [DDS-1], 6-7 points as moderate [DDS-2], and 8-9 points as sufficient [DDS-3] (35).

We defined frequent midnight snacks and social engagement as more than once a week and three times a week, respectively. Sugar-sweetened beverages or coffee overlapped in the first three categories: no, once or twice a week, three to five times a week. Besides, we classified smoking as never, former, current smoking or passive smoking. Alcohol consumption was self-evaluated as “Yes” or “No”. Exercise refers to exercising during leisure time, assessed by whether or not an individual engages in exercise. About sedentary behavior, we evaluated participants based on how many hours they sat one day, including less than two hours, two to four hours, four to six hours, and more than six hours.

### Classification criteria for metabolic syndrome

Waist circumference, systolic and diastolic pressure were collected by trained nurses. The participants were asked to fast for 12 hours before coming to the hospital the next morning for a physical examination. Nurses collected 5 ml of fasting venous blood from the physical examination subjects and distributed it to the clinical laboratory for detection and analysis. The indexes involved in this analysis were fasting blood glucose (FBG), triglyceride (TG) and high-density lipoprotein cholesterol (HDL - C). MetS is a clinical condition characterized by a series of metabolic risk factors. According to the standards of 2013 Chinese Diabetes Society (CDS 2013) (36), which are widely used in Chinese, MetS can be diagnosed if any three or more of the following criteria are met: (1) Abdominal obesity: female waist circumference ≥ 85 cm; (2) Hyperglycemia: FPG ≥ 6.10mmol/L; (3) Hypertension: blood pressure ≥ 130/85 mm Hg and/or diagnosed hypertension and receiving corresponding treatment; (4) TG ≥ 1.70mmol/L; (5) HDL - C < 1.04mmol/L.

### Thyroid diagnosis

Specially trained technicians used color Doppler ultrasonic detector detection, with 7 to 9 MHz probe frequency. The examinee was supine with the neck fully exposed and the characteristics of TN(s) (i.e. nodule number, size, location, echogenicity, central, and peripheral vascularity, calcification and shape) were recorded. A TN was defined as a discrete lesion within the thyroid gland that was radio-logically distinct from the surrounding thyroid parenchyma. Diameter ≥ 3 mm is used to determine the presence of TN(s). According to diagnoses of professional technicians, if the subjects had TN(s), we divided them into the thyroid nodule(s) present group. Others were included in the thyroid nodule(s) absent group. The health management center of the hospital involved in the study provided approval. Participation in the study was entirely voluntary and no reward. Informed consent was obtained from each participant.

In this Chinese hospital, not every participant was required to have thyroid function tests. Based on the ultrasound results, if necessary, thyroid function indicators, including thyroid-stimulating hormone (TSH), triiodothyronine (T3), free triiodothyronine (FT3), thyroxine (T4), free thyroxine (FT4), thyroglobulin antibody (TgAb), and thyroid peroxidase antibody (TPOAb) were determined by an electrochemiluminescence immunoassay.

### Statistical analysis

All collected data were analyzed using SPSS 25.0 software (IBM, Armonk, NY, USA). Categorical variables were expressed by percentages, and comparisons between groups were compared by χ^2^ tests. Fisher’s exact probability method was used to analyze the variables with minimum theoretical frequency between 1 and 5. Not all the people who take part in the physical examination are accepted the thyroid hormone relevant tests. To avoid bias related to missing data, TSH, T3, FT3, T4, FT4, TgAb and TPOAb were excluded due to the large amount of missing data. To adjust for confounding factors, variables with significant inter-group comparison were selected as independent variables, and multilevel logistic regression analysis was used to evaluate the relationship between lifestyle, components of MetS and TN(s). Model 1 only contained demographic characteristics; Model 2 included demographic characteristics and lifestyle. Model 3 was a complete model, and components of MetS were added into the variables of model 2. Odds ratios (OR) and 95% confidence intervals (95% CI) were calculated. Parameters including −2 log likelihood, nagelkerke R^2^, and omnibus χ^2^ were used to compare these multilevel models. P < 0.05 was considered as statistically significant difference.

## Results

### Demographic characteristics and prevalence of thyroid nodules

Totally 2,784 individuals remained enrolled in this study (see **Figure 1**). The prevalence of TN(s) in Chinese adult women was 33.3%. Basic personal characteristics, lifestyle and components of MetS based on whether participants have TN(s) or not are presented in **Table 1**. The study included 933 (33.5%) TN(s) patients and 1851 (66.5%) individuals without TN(s). By age group, women in < 40 years were 27.3%, 29.2% in 40-49 years, 29.7% in 50-59 years and 13.9% in ≥ 60 years. Besides, 59.1% of the participants were brain workers; 61.6% of the participants had normal weight. Only 12.2% of participants had the lowest DDS. More than 90% of the participants did not have frequent midnight snacks (98.3%), frequent social engagement (98.5%), smoking (94.7%) and alcohol consumption (90.7%). Most participants did not have coffee (75.5%) and sugar-sweetened beverages (61.2%). Three-fifths of the participants did not exercise. Almost half of the participants had 2-4 hours per day for sedentary behavior (40.6%). In terms of components of MetS, blood pressure (66.9%), FBG (96.0%), TG (78.2%) and HDL - C (96.4%) were all in the normal range in most participants; only a few participants (10.1%) had central obesity (in **Table 2**).

**Table 1:** Demographic characteristics and lifestyle of all participants with and without thyroid nodules (N = 2,784).

Variables	All Participants	Thyroid nodule (s) absent (n = 1,851)	Thyroid nodule (s) present (n = 933)	χ^2^	P
**1. Demographic characteristics**					
**Age**				112.955	**0.000**
<40 years old	759 (27.3%)	580 (31.3%)	179 (19.2%)		
40-49 years old	813 (29.2%)	590 (31.9%)	223 (23.9%)		
50-59 years old	826 (29.7%)	486 (26.3%)	340 (36.4%)		
≥60 years old	386 (13.9%)	195 (10.5%)	191 (20.5%)		
**Occupations**				79.420	**0.000**
Mainly mental work	1646 (59.1%)	1195 (64.6%)	451 (48.3%)		
Retiree	353 (12.7%)	199 (10.8%)	154 (16.5%)		
Mainly mental and manual work	441 (15.8%)	279 (15.1%)	162 (17.4%)		
Mainly manual work	344 (12.4%)	178 (9.6%)	166 (17.8%)		
**2. Lifestyle**					
**BMI**				18.965	**0.000**
Lean (<18.5 kg/m2)	104 (3.7%)	77 (4.2%)	27 (2.9%)		
Normal weight (18.5-23.9 kg/m2)	1714 (61.6%)	1180 (63.7%)	534 (57.2%)		
Overweight (24.0-27.9 kg/m2)	800 (28.7%)	498 (26.9%)	302 (32.4%)		
Obese (>28.0 kg/m2)	166 (6.0%)	96 (5.2%)	70 (7.5%)		
**DDS**				38.742	**0.000**
DDS-1	339 (12.2%)	211 (11.4%)	128 (13.7%)		
DDS-2	1057 (38.0%)	778 (42.0%)	279 (29.9%)		
DDS-3	1388 (49.9%)	862 (46.6%)	526 (56.4%)		
**Midnight snacks**				2.192	0.139
Yes (>1 times/W)	47 (1.7%)	36 (1.9%)	11 (1.2%)		
No (≤1 time/W)	2737 (98.3%)	1815 (98.1%)	922 (98.8%)		
**Social engagement**				3.312	0.069
Yes (>3 times/W)	43 (1.5%)	1828 (98.8%)	913 (97.9%)		
No (≤3 times/W)	2741 (98.5%)	23 (1.2%)	20 (2.1%)		
**Coffee**				14.350	**0.002**
No	2102 (75.5%)	1359 (73.4%)	743 (79.6%)		
1-2 times/W	554 (19.9%)	394 (21.3%)	160 (17.1%)		
3-5 times/W	80 (2.9%)	61 (3.3%)	19 (2.0%)		
Every day	48 (1.7%)	37 (2.0%)	11 (1.2%)		
**Sugar-sweetened beverages**				0.989	0.610
No	1705 (61.2%)	1140 (61.6%)	565 (60.6%)		
Sometimes (1-2 times/W)	1023 (36.7%)	677 (36.6%)	346 (37.1%)		
Often (3-5 times/W)	56 (2.0%)	34 (1.8%)	22 (2.4%)		
**Smoking**				1.940	0.587
Never	2636 (94.7%)	1751 (94.6%)	885 (94.9%)		
Former	11 (0.4%)	7 (0.4%)	4 (0.4%)		
Current	61 (2.2%)	45 (2.4%)	16 (1.7%)		
Passive-smoker	76 (2.7%)	48 (2.6%)	28 (3.0%)		
**Alcohol consumption**				0.028	0.868
Yes	259 (9.3%)	171 (9.2%)	88 (9.4%)		
No	2525 (90.7%)	1680 (90.8%)	845 (90.6%)		
**Exercise**				4.790	**0.029**
Yes	1097 (39.4%)	756 (40.8%)	341 (36.5%)		
No	1687 (60.6%)	1095 (59.2%)	592 (63.5%)		
**Sedentary behavior**				2.009	0.570
<2 hours/day	831 (29.8%)	550 (29.7%)	281 (30.1%)		
2-4 hours/day	1131 (40.6%)	747 (40.4%)	384 (41.2%)		
4-6 hours/day	524 (18.8%)	345 (18.6%)	179 (19.2%)		
>6 hours/day	298 (10.7%)	209 (11.3%)	89 (9.5%)		

p values were from the chi-square test for categorical variables; Categorical variables are presented as number (percentage). BMI, Body mass index; DDS, Dietary diversity scale.Bold means, P values are statistically significant.

**Table 2:** Components of Metabolic syndrome among all participants with and without thyroid nodules (N = 2,784).

Variables	All Participants	Thyroid nodule (s) absent (n = 1,851)	Thyroid nodule (s) present (n = 933)	χ^2^	P
**Hypertension**				29.821	**0.000**
Yes	922 (33.1%)	549 (29.7%)	373 (40.0%)		
No	1862 (66.9%)	1302 (70.3%)	560 (60.0%)		
**Central obesity**				25.857	**0.000**
Yes	280 (10.1%)	224 (12.1%)	180 (19.3%)		
No	2504 (89.9%)	1627 (87.9%)	753 (80.7%)		
**Level of FBG**				11.284	**0.001**
Hyperglycemia (FBG ≥6.1 mmol/L)	111 (4.0%)	161 (8.7%)	119 (12.8%)		
Normal	2673 (96.0%)	1690 (91.3%)	814 (87.2%)		
**Level of TG**				32.854	**0.000**
High TG (≥1.70 mmol/L)	606 (21.8%)	344 (18.6%)	262 (28.1%)		
Normal	2178 (78.2%)	1507 (81.4%)	671 (71.9%)		
**Level of HDL -C**				0.061	0.805
Low HDL -C (<1.04 mmol/L)	101 (3.6%)	66 (3.6%)	35 (3.8%)		
Normal	2683 (96.4%)	1785 (96.4%)	898 (96.2%)		

p values were from the chi-square test for categorical variables; Categorical variables are presented as number (percentage); Hypertension: blood pressure ≥ 130/85 mm Hg and/or diagnosed hypertension; Abdominal obesity: female waist circumference ≥ 85 cm; Hyperglycemia (FBG ≥ 6.1 mmol/L). FBG, fasting blood glucose; TG, triglyceride; HDL -C, High-density lipoprotein cholesterol; LDL -C, Low-density lipoprotein cholesterol.Bold means, P values are statistically significant.

### Bivariate analysis of thyroid nodules

As shown in **Table 1**, subjects with TN(s) were significantly older, and women in 50-59 years old (36.4%) had a much higher prevalence than 40-49 years old (23.9%) and < 40 years old (19.2%). They were more likely to be brain worker and become overweight than participants without TN(s) (P < 0.001). According to dietary habits, TN participants showed a higher percentage of DDS-1 and a lower percentage of DDS-2 (P < 0.001). Except for coffee and exercise (P < 0.001), the two groups had no significant differences in midnight snacks, social engagement, sugar-sweetened beverages, smoking, alcohol consumption and sedentary behavior. About components of MetS, the two groups in hypertension, central obesity, high levels of FBG and TG differed significantly (P < 0.05).

### Multilevel logistic regression analysis

To adjust for confounding factors, Model 1 included only demographic characteristics; Model 2 added lifestyle to Model 1; Model 3 was a complete model that included demographic characteristics, lifestyle, and components of the metabolic syndrome. As shown in **Table 2**, parameter −2 log likelihood became smaller and smaller, while nagelkerke R^2^ and omnibus χ^2^ gradually increased, indicating that model 3 provided the best prediction. In the full model (Model 3, **Table 2**), women in 50-59 years old (odds ratio (OR) [95% confidence interval (95% CI)]): 1.746 [1.356-2.249] and older than 60 (2.147 [1.540-2.993]) were more likely to have a risk of TN(s) (P < 0.001). With the increase of manual work, subjects were at progressively higher risk of TN(s): women in mainly mental and manual work (1.465 [1.163-1.845]) and in mainly manual work (1.780 [1.367-2.317]) showed a higher risk than mental work (P < 0.05). In a certain range, DDS-2 (0.624 [0.476-0.817]) significantly reduced the risk of TN(s) occurrence compared with DDS-1 (P < 0.001). Besides, low TG level (0.739 [0.604-0.905]) was a protective factor for TN(s) (P < 0.05). Finally, −2 log likelihood of model 3 = 3,352.286, nagelkerke R^2^ = 0.096, and omnibus χ^2 =^ 198.716 for Model 3 (in **Table 3**).

**Table 3:** Multilevel logistic regression analysis of the relationship between thyroid nodules and individual characteristics, lifestyle, and components of metabolic syndrome (N = 2,784).

Variables	Odds Ratio [95% Confidence Interval]		
	Model 1	Model 2	Model 3
**1. Demographic characteristics**			
**Age**			
<40 years old	1.000	1.000	1.000
40-49 years old	1.216 [0.965-1.531]	1.155 [0.909-1.468]	1.112 [0.873-1.417]
50-59 years old	2.083*** [1.653-2.626]	1.915*** [1.499-2.446]	1.746*** [1.356-2.249]
≥60 years old	2.720*** [2.014-3.675]	2.459*** [1.793-3.372]	2.147*** [1.540-2.993]
**Occupations**			
Mainly mental work	1.000	1.000	1.000
Retiree	1.194 [0.906-1.575]	1.188 [0.899-1.570]	1.184 [0.895-1.566]
Mainly mental and manual work	1.479*** [1.179-1.855]	1.481** [1.177-1.864]	1.465* [1.163-1.845]
Mainly manual work	1.778*** [1.380-2.292]	1.796*** [1.381-2.335]	1.780** [1.367-2.317]
**2. Lifestyle**			
**BMI**			
Lean (<18.5 kg/m^2^)		1.000	1.000
Normal weight (18.5-23.9 kg/m^2^)		1.102 [0.687-1.768]	1.058 [0.659-1.697]
Overweight (24.0-27.9 kg/m^2^)		1.228 [0.753-2.004]	1.034 [0.626-1.707]
Obese (>28.0 kg/m^2^)		1.336 [0.758-2.355]	0.939 [0.500-1.764]
**DDS**			
DDS-1		1.000	1.000
DDS-2		0.624** [0.477-0.816]	0.624** [0.476-0.817]
DDS-3		1.086 [0.840-1.404]	1.095 [0.846-1.418]
**Coffee**			
No		1.000	1.000
1-2 times/W		0.864 [0.697-1.071]	0.871 [0.702-1.080]
3-5 times/W		0.707 [0.414-1.208]	0.731 [0.428-1.249]
Every day		0.721 [0.361-1.440]	0.729 [0.364-1.460]
**Exercise**			
Yes		1.000	1.000
No		1.106 [0.928-1.319]	1.122 [0.940-1.339]
**3. Components of MetS**			
**Hypertension**			
Yes			1.000
No			0.875 [0.727-1.052]
**Central obesity**			
Yes			1.000
No			0.789 [0.586-1.062]
**Level of FBG**			
High FBG (≥6.1 mmol/L)			1.000
Normal			1.034 [0.783-1.364]
**Level of TG**			
High TG (≥1.70 mmol/L)			1.000
Normal			0.739** [0.604-0.905]
**−2 log likelihood**	3412.675	3366.021	3352.286
**Nagelkerke R^2^**	0.067	0.089	0.096
**Omnibus χ^2^**	138.327	184.981	198.716

Model 1: adjusted for age, occupations; Model 2: adjusted for adjusted for age, occupations, BMI, DDS, coffee, exercise; Model 3: adjusted for age, occupations, BMI, DDS, coffee, exercise, hypertension, central obesity, level of FBG and level of TG. *p < 0.05; **p < 0.01; ***p < 0.001.

## Discussion

The purpose of this cross-sectional study was to determine the impact of lifestyle (dietary habits, excise and sedentary behavior), components of MetS on TN(s). In our study, the prevalence of TN(s) was 33.3% in Chinese adult women, slightly lower than in all Chinese females (37), and was almost consistent with adult women in sub-Saharan setting (38). Differences may be due to age, genetics, living environment, dietary habits and iodine nutrition status.

Age and occupations were two independent risk factors for TN(s). The average age of patients with TN(s) was higher than that without TN(s), mainly over 50 years old, indicating that the prevalence of TN(s) increased significantly with age, which was consistent with most research results (5, 39). This may be related to the decline in thyroid function caused by aging. As people grow older, thyroid tissue will undergo fibrosis, cell infiltration and follicular changes, eventually leading to the formation of nodules (40). Besides, the prevalence of breast nodules is also greater in old women (41). Although the coincidence of thyroid and breast diseases remains controversial, two recent articles found thyroid nodules coexist with either cystic or solid breast nodules (42, 43). We will analyze breast nodules in the future to provide in-depth support for this study as much as possible. Therefore, attention and screening for TN(s) should be strengthened in older women, especially those over 50 years old.

The relative risk of TN(s) for women in mainly manual work and in mainly mental and manual work were one point eight and one point five times that of brain workers, respectively. This suggests that the increased prevalence of TN(s) is associated with an increase in physical labor intensity at work. A study showed that in the population suffering from thyroid related diseases, the proportion of patients who mainly work physically was significantly higher than those who mainly work mentally (44). In contrast, a cross-sectional study found that the prevalence of TN(s) increased as physical labor and physical activity intensity decreases, but this association did not exist in women (5). Other studies showed that labor and exercise were not independent factors in the development of TN(s) through multifactorial adjustment. Besides, perhaps these differences are likely due to manual workers have greater likelihood of exposure to pollutants, such as workers in petrochemical plants, aluminum manufacturers, etc (45). And other metabolic factors associated with labor and exercise may be involved in the development of TN(s). It is suggested that future studies should also further evaluate the intensity of manual work and determine its association with pollutants.

Dietary diversity is a proxy for micro-nutrient adequacy in diets (46), and iodine deficiency or adequacy has been recognized as an independent risk factor for thyroid disease. The risk of goiter in DDS-1 was almost one point six times higher than in DDS-2, probably because of the low availability of iodine when women do not have access to a diverse diet (47). In some areas with monotonous grain-based diets, most people suffer from thyroid disease and the presence of micro-nutrient deficiencies such as vitamin A and iron deficiency (48). A cross-sectional study in Ethiopia also showed that patients with poor DDS had a higher incidence of thyroid disease (18, 47). A Connecticut case-control study observed a negative association between a diet high in fruits and vegetables and the risk of TC (49), consistent with studies in Greece and Poland (50, 51). It can be seen that dietary diversity is significantly correlated with thyroid disease. The aforementioned dietary pattern based on vegetables, fruits, and whole grains rather than meat was found to have a stronger protective effect in women, especially those aged 50 or older (49). In contrast, one study suggested that the higher the total energy ratio of fat to saturated fat and cholesterol intake, the more varied the diet, especially in poor rural Mexico (52). This difference may be related to the health perception and economic level of the subjects. It must be admitted that the participants in this study are almost a group of people with a fixed income, because they can take the initiative to go to the hospital or have a physical examination organized by the unit. Therefore, future studies should specifically analyze the impact of dietary diversity on health according to individual health cognition and economic status, and pay attention to food attributes and diet balance. Some studies have also found an increase in thyroid disease in people exposed to polluted environmental and food, such as petrochemical workers, areas contaminated with organochlorine pesticides or polychlorinated biphenyls, or near aluminum foundries (45, 53). Fish (particularly the large, top-predator fish like swordfish) from polluted waters have more likely high levels of mercury. Mercury was found to be a known autoimmune environmental trigger and can be measured inside the thyroid (54). Fortunately, nutrients such as omega-3 fatty acids and selenium found in fish have been shown to exert a protective effect against thyroid disease and reduced the risk of cancer (53). Therefore, future research should not ignore the possibility of food contamination.

The importance of lifestyle factors has paid more and more attention in thyroid nodule prevention (5, 55, 56). Smoking and alcohol consumption are closely related to the prevalence of goiter (6). Surprisingly, our data did not confirm that smoking and alcohol use were significantly different between the groups. Perhaps our survey included women who hardly ever smoked or drank alcohol, and did not include men. This difference may be closely related to the fact that few studies have looked at women alone. Future multiple separate studies of women are needed to test the current findings.

Little researches have been done on the potential effects of exercise on thyroid function. We found exercise was not an independent predictor of TN(s), and no difference in sedentary behavior between the two groups. A cross-sectional study, which did not observe such an association in women, showed that labor and exercise were also not independent factors in the development of TN(s) through multi-factor adjustment (5). In contrast, one cohort study suggested that physical activity was negatively correlated with goitre and serum thyroglobulin (57). Perhaps these differences are due to other metabolic factors associated with labor and exercise that may be involved in TN(s) development.

Metabolic Syndrome is indeed associated with the prevalence of TN(s). Component diseases such as central obesity, abnormal blood pressure, hyperglycemia, and hypertriglyceridemia are also associated with thyroid nodules, a finding supported by several meta-analyses (25, 29). However, our results did not show a correlation between low HDL - C and thyroid nodules, which is inconsistent with other conclusions (58). This may be caused by a small portion (3.6%) of the lower HDL - C in this study. No deference could be detected on TN(s) between the women with lower HDL - C and those with normal HDL - C. Future studies with a comparable ratios of women with lower HDL -C and normal HDL - C will be conducted to further explore the relationship of TN(s) and HDL - C. In addition, high triglyceride levels are also a risk factor for TN(s), which is consistent with meta-analysis and cross-sectional studies of urban populations in southwest China (29, 59). May be hyperlipidemia stimulates thyroid cell hyperplasia and angiogenesis to trigger thyroid nodules (58), and modern industrialization has brought about overnutrition and a sedentary lifestyle.

In conclusion, the prevalence of TN(s) in Chinese adult women was 33.3%. Age over 50 years old and non-brain worker were risk factors for TN(s), while higher DDS degrees and low level of triglyceride were protective factors. Adult women, especially those over 50 and their jobs are mainly manual should enhance screening, follow-up and health management of TN(s). Higher dietary diversity is protective measures against TN(s). It is important to note that a rich diet should focus on food attributes and a balanced diet rather than increasing quantities. The combined pattern of diet and exercise will have a significant impact, which combine to create a healthy internal host environment or metabolic state.

Limitations of the current study. First, this was a cross-sectional study, and the results cannot be used to predict the development of TN(s). Future prospective cohort studies are indispensable. Although the participants in this study came from one hospital in southern China, we still need to expand sample sources to different regions. Second, we only assessed whether the participants had TN(s), without distinguishing the size and number of nodule(s), which is significant for further researches on the correlation between the lifestyle of patients with TN(s) and the size and number of nodules. In addition, we acknowledged the lack of thyroid function tests, menstrual history and iodine nutritional status, which would have provided more in-depth support of our findings. Finally, although the coincidence of thyroid and breast diseases remains controversial, a recent article found thyroid nodules coexist with either cystic or solid breast nodules. We will analyze breast nodules in the future to provide in-depth support for this study as much as possible.

## Data availability statement

The original contributions presented in the study are included in the article/supplementary material. Further inquiries can be directed to the corresponding authors.

## Ethics statement

The studies involving human participants were reviewed and approved by Ethics Committee of The Third Xiangya Hospital of Central South University. The patients/participants provided their written informed consent to participate in this study.

## Author contributions

XD and YLi contributed equally to this work. Conceptualization, XD and YLi; methodology, YD; software, LL; validation, LL, ZW, YK and YLi; formal analysis, XD and YLi; investigation, YLi and JW; resources, YLi and JW; data curation, XD and YLi; writing-original draft preparation, XD; writing-review and editing, XD, YLi, YD, JX, LL, SD and AC; visualization, ZW, YK and YLou; supervision, YD; project administration, JX and YD; funding acquisition, YD. All authors contributed to the article and approved the submitted version.

## Funding

This work was supported by the Special Funding for the Construction of Innovative Provinces in Hunan(No.2020SK53618).

## Conflict of interest

The authors declare that the research was conducted in the absence of any commercial or financial relationships that could be construed as a potential conflict of interest.

## Publisher’s note

All claims expressed in this article are solely those of the authors and do not necessarily represent those of their affiliated organizations, or those of the publisher, the editors and the reviewers. Any product that may be evaluated in this article, or claim that may be made by its manufacturer, is not guaranteed or endorsed by the publisher.
